# Tall stature: a difficult diagnosis?

**DOI:** 10.1186/s13052-017-0385-5

**Published:** 2017-08-03

**Authors:** Cristina Meazza, Chiara Gertosio, Roberta Giacchero, Sara Pagani, Mauro Bozzola

**Affiliations:** 1 Department of Internal Medicine and Therapeutics, Unit of Pediatrics and Adolescentology, University of Pavia, Fondazione IRCCS Policlinico San Matteo, Piazzale C. Golgi 19, 27100 Pavia, Italy; 2 University of Pavia, Fondazione IRCCS Policlinico San Matteo, Pavia, Italy; 3 Department of Pediatrics, San Paolo Hospital, Milan, Italy

**Keywords:** Tall stature, Syndromes, Growth velocity, Puberty, Height

## Abstract

Referral for an assessment of tall stature is less common than for short stature. Tall stature is defined as a height more than two standard deviations above the mean for age. The majority of subjects with tall stature show a familial tall stature or a constitutional advance of growth (CAG), which is a diagnosis of exclusion. After a careful physical evaluation, tall subjects may be divided into two groups: tall subjects with normal appearance and tall subjects with abnormal appearance. In the case of normal appearance, the paediatric endocrinologist will have to evaluate the growth rate. If it is normal for age and sex, the subject may be classified as having familial tall stature, CAG or obese subject, while if the growth rate is increased it is essential to evaluate pubertal status and thyroid status. Tall subjects with abnormal appearance and dysmorphisms can be classified into those with proportionate and disproportionate syndromes.

A careful physical examination and an evaluation of growth pattern are required before starting further investigations. Physicians should always search for a pathological cause of tall stature, although the majority of children are healthy and they generally do not need treatment to cease growth progression.

The most accepted and effective treatment for an excessive height prediction is inducing puberty early and leading to a complete fusion of the epiphyses and achievement of final height, using testosterone in males and oestrogens in females. Alternatively, the most common surgical procedure for reducing growth is bilateral percutaneous epiphysiodesis of the distal femur and proximal tibia and fibula.

This review aims to provide up-to-date information and suggestions about the diagnosis and management of children with tall stature.

## Background

Human growth is a complex and dynamic physiological process tightly regulated by genetic, hormonal, nutritional and environmental factors. It is characterised by somatic changes in stature, body proportion, and body composition that involve cell hyperplasia (increase in cell number), cell hypertrophy (increase in cell size) and apoptosis (programmed cell death) [1].

Growth can be considered to occur in four separate but closely integrated phases, according to dominant influence from different factors. The first of these is the intrauterine phase, dependent upon maternal factors, nutrition and placental function, and coordinated by growth-promoting factors. The second is the infancy phase of growth, occurring mainly during the first 2–3 years of post-natal life; this period is driven by nutritional factors. The third phase of growth is the childhood phase, during which growth hormone (GH) and thyroid hormones become important in regulating growth. Finally, the pubertal growth spurt is controlled by the synergistic action of GH and sex steroids. [1].

An individual’s potential for growth is influenced by a number of genetic, environmental and hormonal factors.

Children with tall stature are rarely referred to a paediatric endocrinologist for investigations about their condition, much less often than a referral for short stature. However, although most tall children are healthy but there are pathological causes of tall stature, paediatricians should always consider this eventuality [2]. In fact, some syndromes with severe complications are associated with abnormal body measurements including sitting height and arm span. However, a thorough history including information on birth, physical examination and evaluation of the growth chart may suffice to conclude whether there is a benign cause for tall stature. For example, increased birth weight and length are observed in Beckwith-Wiedemann syndrome, while developmental problems may be associated with Klinefelter, Triple X, fragile X, homocystinuria, Sotos and Weaver syndrome. Furthermore, an accurate medical history may reveal a syndromic cause: lens luxation may suggest homocystinuria or Marfan syndrome, cardiovascular problems are typical of Marfan syndrome, neonatal hypotonia is present in Sotos syndrome and abdominal wall and macroglossia defects can be seen in Beckwith-Wiedemann syndrome. A careful history and a precise physical examination are essential in order to point towards the cause of tall stature and to decide which diagnostic tests are necessary to reach a diagnosis.

An X-ray of the non-dominant hand should be used to evaluate the skeletal maturity of tall children and it is useful to predict adult height. The available methods to predict adult height are, however, imperfect as Bayley-Pinneau method overestimate adult height and Tanner-Whitehouse Mark 1 and 2 overestimate or underestimate it depending on bone age [3, 4].

Finally, in our opinion, it is important to follow-up tall subjects since some epidemiological studies have shown that taller people are at increased risk of common cancer such as breast, ovary, prostate and large bowel [5, 6]. In particular, a British study showed that tall women have a greater risk of developing breast, endometrium, colon and ovarian cancer [7] and another Swedish study found that total cancer risk and risks of breast cancer and melanoma were higher with increasing height [8].

### Management of a child with tall stature

Firstly, tall stature is defined as a height more than two standard deviations (SD) above the mean for age, i.e. greater than the 97th percentile for sex and age. It is important to take into account the ethnic background of the patient and use the appropriate growth charts when measuring a child. Secondly, a child can also be considered tall in relation to his/her midparental height (MPH), when his/her height is more than two SD over the MPH (height standard deviation score (SDS)-MPH SDS >2.0). Therefore, a detailed familial history of tallness in one or both parents is essential in order to not miss a diagnosis.

**Fig. 1:**
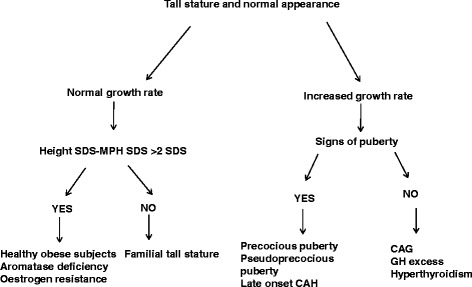
Diagnostic approach to tall children with normal appearance. MPH: midparental height, SDS: standard deviation score, CAG: constitutional advance of growth, GH: growth hormone, CAH: congenital adrenal hyperplasia

**Fig. 2:**
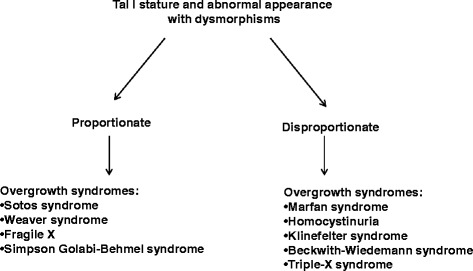
Diagnostic approach to tall children with dysmorphisms

After a careful physical evaluation, tall subjects may be divided into two groups (Figs. 1 and 2): tall subjects with normal appearance and tall subjects with abnormal appearance.

In the case of normal appearance (Fig. 1), the paediatric endocrinologist will have to evaluate growth rate. If growth velocity is normal for age and sex, the subject grows according to his/her MPH and his/her body mass index (BMI) is within the normal ranges, he may be classified as ***familial tall stature*** and a family history of tallness in one or both parents should have been previously reported. The height of these subjects is usually within the target centile range calculated from the parents’ height [9]. No further investigation is required as long as the child normally develops and has a normal physical examination.

On the contrary, if the difference height SDS-MPH SDS is >2 and the BMI is above the normal range (>95th percentile), the subject has to be classified as ***obese***. Obese children are sometimes taller than average for their age and, if maturity is also advanced, puberty may occasionally occur earlier [10]. However, their final height will usually be normal.

If the BMI is normal an ***aromatase deficiency*** may be suspected. This is a rare disease with autosomal recessive inheritance. In males the diagnosis is made later in adulthood, when there is a tall stature, incomplete epiphyseal closure, eunucoid proportion of the skeleton, osteoporosis and obesity. Oestrogen levels are low, while follicle stimulating hormone (FSH), luteinizing hormone (LH) and testosterone increased slightly. The most intriguing features are the presence of steatohepatitis, insulin resistance with acanthosis nigricans and high concentrations of triglycerides. The administration of low-dose oestrogen allows complete bone maturation after the complete closure of the epiphysis and produces an increase in bone density. More than 30 mutations (point mutations, deletions and insertions) have been found in the gene *CYP19A1*, which encodes for the human P450 aromatase enzyme located in the membrane of the endoplasmic reticulum of several tissues, such as gonad, brain, placental syncytiotrophoblast, breast, and adipose tissue and catalyzes biosynthesis of oestrogens from androgens [11].

Rare cases of ***oestrogen receptor*** *α* ***(ER*** *α* ***) deficiency*** have been described and the phenotype is very similar to that of aromatase deficiency with tall stature and eunucoid body proportions, continued linear growth into adulthood due to incomplete epiphyseal closure and osteoporosis in males [12] and absent breast development and markedly elevated serum oestrogen levels and multicystic ovaries in females [13]. Both oestrogen-resistant patients had homozygous point mutations in the *ERα* gene [12, 13].

When an increase in growth velocity is observed, it is essential to evaluate pubertal status and thyroid status. In fact, tall subjects with normal appearance and high growth velocity may be divided into two groups according to the absence or presence of pubertal signs (Fig. 1).

If there are pubertal signs, subjects may show ***precocious puberty*** in the presence of the appearance of the breast (Tanner stage 2) before 8 years in females and enlargement of testicular volume (>4 ml) before the age of 9 years in males, in both associated with advanced bone age. Gonadotropin releasing hormone (GnRH) stimulation test is necessary to confirm the diagnosis of precocious puberty and therapy with GnRH analogues should be promptly started in order to block pituitary gonadotropin secretion. The presence of pubertal signs may also suggest an early ***pseudoprecocious puberty*** with high steroid, androgen or oestrogen secretion, secondary to an ovarian cyst in females or, more rarely to testis or ovary cancer, or a ***congenital adrenal hyperplasia.*** Congenital adrenal hyperplasia is mainly (90% of cases) due to a 21-hydroxylase deficit encoded by the gene *GYP21A2* and tall stature, premature pubarche, irregular menstruation, acne, hirsutism and bone age advancement characterize the late-onset form of this condition.

Tall children without pubertal signs and increase of growth rate include children with ***constitutional advance of growth (CAG)***. They present an accelerated growth after birth reaching peak centile by 2–4 years of age, and then they grow along 97th centile until 9 year when their growth rate drops to 50th percentile [14, 15]. These children are different from those with familial tall stature since they are born with an above average birth length and they are tall for their parental height [2, 16]. Sometimes CAG can be associated with obesity and it has been suggested that could predict late onset of childhood obesity in non-obese children [16]. Puberty is often earlier in these children. An increase in insulin-like growth factor (IGF)-II secretion and a higher IGFs/IGF binding proteins (BPs) molar ratio prior to puberty may be a possible mechanism of tall stature in CAG children [17].

In the absence of pubertal signs associated with a growth rate increase a condition of ***GH excess*** or ***hyperthyroidism*** may be considered. The former occurs before epiphyseal fusion and is characterized by rapid growth not associated with advanced bone age, metabolic changes similar to those observed in acromegalic patients and elevated serum IGF-I values. Furthermore, serum GH values are not suppressed by glucose oral administration. Since a GH-secreting tumour may be present, radiologic evaluation of the hypothalamus and pituitary by magnetic resonance imaging is requested. In a condition of hyperthyroidism, an increase in growth rate associated with advanced bone age is present. The diagnosis is made by measuring free thyroxine and thyroid stimulating hormone (TSH) levels.

***McCune-Albright Syndrome (MAS)*** is a rare cause of precocious pseudopuberty and GH excess. It is a genetic disorder originally characterized as the triad of polyostotic fibrous dysplasia of bone, precocious puberty and café-au-lait skin pigmentation. Moreover, other associated endocrinopathies have been recognized, including hyperthyroidism, GH excess, FGF23-mediated phosphate wasting and hypercortisolism. Skin manifestations are common and are usually present at or shortly after birth. The café-au-lait spots typically have irregular margins giving them a “coast of Maine” appearance, and usually show an association with the midline of the body. In MAS, fibrous dysplasia of bone typically occurs at several sites (polyostotic), and commonly presents with fracture, deformity and/or bone pain. Radiographs show characteristic expansile lesions with a “ground glass” appearance. Craniofacial fibrous dysplasia can be severe in individuals who have pituitary disorders leading to GH hypersecretion. In girls, precocious pseudopuberty is a common initial manifestation, with recurrent ovarian cysts leading to episodes of vaginal bleeding and breast development, while it is less common in boys, presenting with penile enlargement, pubic and axillary hair, acne, body odour, and sexual behaviour. However, in both girls and boys, there is a high frequency of gonadal pathology (ovarian abnormalities in girls, and testicular abnormalities in boys) [18]. McCune-Albright Syndrome is caused by an activating mutation in the *GNAS* gene, which encodes the alpha subunit of the stimulatory G protein involved in G-protein signalling. The mutation arises early in embryogenesis and is distributed in a mosaic pattern.

In many syndromes with dysmorphisms, severe complications may be associated with tall stature. Anthropometric measurement such as height, weight, BMI, arm span, sitting height and head circumference allow a classification of proportionate and disproportionate tall stature, although this cannot be an absolute division (Fig. 2). Physical examination can focus on dysmorphic features including supernumerary nipples such as in Simpson-Golabi-Behmel syndrome or macroglossia such as in Beckwith-Wiedemann syndrome.

### Proportionate syndromes

***Sotos syndrome*** (cerebral gigantism): children with this syndrome at birth have increased length, weight and head circumference [2]. They are large-for-gestational age infants and have rapid growth in early childhood with advanced bone age, but do not attain excessively tall adult height. Characteristic features include a large dolichocephaly, macrocephaly, prominent forehead, down-slanting palpebral fissures, mild hypertelorism, high-arched palate, prominent ears, jaw and small pointed chin, large hands and feet, and acromegalic appearance. Most have subnormal intelligence and motor incoordination. Although they continue to rapidly grow during the early years of life, puberty is early and causes premature epiphyseal fusion leading to normal final height. They have normal GH and IGF-I secretion, thyroid, adrenal and gonadal function. Deletions and point mutations in the *NSD1* gene, with consequent loss of function, account for the majority of cases and there may be autosomal dominant transmission.

***Weaver syndrome*** subjects present prenatal and postnatal overgrowth, typical features including large ears, depressed nasal bridge, downslating palpebral fissures, dimpled chin, prominent wide philtrum and micrognathia. Advanced skeletal maturation and camptodactyly are observed. Many patients present developmental delay and learning difficulties. Missense and truncating mutation in the *EZH2* gene, encoding for histone methyltransferase, are reported [19].

***Fragile X syndrome*** is a genetic disorder that includes intellectual disability and features such as a long and narrow face, protruding ears, flexible fingers, hypotonia and large testicles (macroorchidism). These patients show increased growth velocity in peripubertal period, but it decreases after puberty. The patients often present autism, delayed speech and hyperactivity. Fragile X syndrome occurs in about 1 in 4000 males and 1 in 8000 females. Partial or complete loss-of-function mutations in the *FMR1* gene, mapped on chromosome Xq27.3, cause fragile X syndrome. Most affected patients show hypermethylated CGG-repeat stretch in the 50-untranslated region of the *FMR1* exon 1 [20]. This hypermethylation results in constriction of the X chromosome which appears ‘fragile’ under the microscope at that point, a phenomenon that gave the syndrome its name. Fragile X syndrome has traditionally been considered an X-linked dominant condition with variable expressivity. Prenatal testing with chorionic villous sampling or amniocentesis allows diagnosis of *FMR1* mutations while the foetus is in uterus and appears to be reliable [21].

***Simpson-Golabi-Behmel syndrome*** is a rare inherited congenital disorder that can cause craniofacial, skeletal, cardiac and renal abnormalities. Possible symptoms and associated conditions are macrosomia, macroglossia, advanced bone age, organomegaly, neonatal hypoglycaemia, congenital diaphragmatic hernia, “bulldog” or “coarse” face, short and broad hands and feet with dysplastic nails, polydactyly, pectus excavatum, talipes, vertebral segmentation defects, hypernumerary nipples, structural and conductive cardiac defects, multicystic dysplastic kidneys, hypotonia, brain malformations, developmental disabilities and, rarely, mental retardation. The syndrome is inherited in an X-linked recessive fashion, where males express the phenotype and females usually do not or may express varying degrees of the phenotype. The first signs of Simpson-Golabi-Behmel syndrome may be observed as early as 16 weeks of gestation [22, 23]. These patients show an increased risk of neoplasms, in particular in the abdominal region, such as Wilms tumour and hepatoblastoma. In Simpson-Golabi-Behmel syndrome type I patients, a mutation of the *GPC3* gene (especially expressed in kidney, liver and lung) on the X chromosome locus q26.1 has been described, while Simpson-Golabi-Behmel syndrome type II may be caused by duplication of the *GPC4* gene, which helps to regulate cell division and growth.

### Disproportionate syndromes

***Marfan syndrome*** is an autosomal-dominant condition resulting from a mutation in the *FBN-1* gene on chromosome 15q, encoding for a 350-kDa extracellular matrix glycoprotein that takes part in the formation of microfibrils, which are important for elasticity and structural support of numerous tissues [24]. It affects approximately 1 in 5000 people. They have long limbs with narrow hands and long slender fingers. Arm span is greater than height and the lower segment is much greater than the upper segment. Other features include mild hyperextensible joints, skin striae, kyphoscoloiosis, deformities of the rib cage, high arch palate and lens dislocation. Aortic root dilatation, aortic aneurysms, and mitral valve prolapse are important cardiac features and associated with increased mortality in early adult life. Death from a dissecting aneurysm may occur in early adult life. Echographic and ophthalmic assessment should be undertaken in a child with marfanoid features. Two important clinical findings in making the diagnosis are the wrist sign and thumb sign. The “wrist sign” is positive when the thumb overlaps the fifth finger when grasping the contralateral wrist. The “thumb sign” is positive when the thumb extends well beyond the ulnar border of the hand when overlapped by the fingers [25].

***Homocystinuria*** is a rare (1:250,000) autosomal-recessive disorder caused by an absence of the enzyme cystathionine β-synthase (CBS). Phenotypically, patients resemble those with Marfan syndrome, but they usually have subnormal intelligence, osteopoenia, and a tendency to fatal thromboembolism. Lenticular dislocation also occurs, usually in downward direction. A multitude of genes are related to homocystinuria, but mutations in the gene that encodes for CBS are the prevalent. Alterations in CBS result in the disruption of enzyme activity, which consequently leads to elevated homocysteine, a potentially toxic aminoacid responsible for clinical manifestations observed in homocystinuric patients. To date, 150 *CBS* mutations have been identified and 67% are missense mutations [26].

***Klinefelter syndrome*** has a frequency of around 1 in 1000 live born males, and the incidence increases with maternal age. Most have the 47, XXY kariotype, and about 10% are mosaicism (i.e. 47,XXY/46,XY). In childhood, presentation is usually with tall stature and poorly developed secondary sexual characteristics, with adults presenting with infertility. Patients tend to be tall with disproportionately long limbs, feminine distribution of body fat, gynaecomastia and mild learning difficulties. Onset of puberty is not delayed, but testicular volume does not increase more than 8-10 ml. Testicular histology shows seminiferous dysgenesis, increase in Leydig cells and interstitial fibrosis. Subjects with Klinefelter syndrome may develop breast cancer or metabolic syndrome.

***Beckwith-Wiedemann syndrome*** is associated with excessive overgrowth apparently caused by excess availability of IGF-II. It is characterized by foetal macrosomia with omphalocele, macroglossia, renal medullary hyperplasia, and neonatal hypoglycaemia due to islet cell hyperplasia. No abnormality of the GH-IGF axis has been identified. A cluster of imprinted genes associated with BWS is present at chromosome 11p15.5.

***Triple X (XXX) syndrome*** **.** The incidence of this syndrome is 1 in 1000 birth females, but the majority are not recognized due to considerable variation in the phenotype [27]. About 10% of subjects may be mosaicisms (i.e. 46 XX/47 XXX, 47 XXX/48 XXXX, 45 X/47 XXX, 45 X/46 XX/47 XXX). Features include increased linear growth in mid-childhood, tall stature, epicanthal folds, hypertelorism, upslanting palpebral fissures, clinodactily, hypotonia, joint hyperextensibility, genitourinary abnormalities, congenital hip dysplasia, premature ovarian failure, congenital heart defects, seizure disorders and electroencephalogram abnormalities. Fertility is normal. Increased risk for attention deficit and early developmental delays, mainly in speech-language disorders may be present.

Other rare conditions are characterized by tall stature:

***Proteus syndrome*** is mainly caused by activating mutations of *AKT1* and *PTEN* genes and the characteristic signs are macrocephaly with frontotemporal exostosis, large hands and feet with macrodactyly, normal psychomotor development, predisposition to cancer and thromboembolic diseases.

***Berardinelli-Seip congenital lipodystrophy*** is usually diagnosed at birth or soon thereafter. Because of the absence of functional adipocytes, lipid is stored in other tissues, including muscle and liver. Affected individuals develop insulin resistance and approximately 25–35% develop diabetes mellitus between ages 15 and 20 years. Hepatomegaly secondary to hepatic steatosis and skeletal muscle hypertrophy occur in all affected individuals. Hypertrophic cardiomyopathy is reported in 20–25% of affected individuals and it is a significant cause of morbidity for cardiac failure and early mortality.

In conclusion, to investigate a syndromic subject with tall stature, the following assessments should be initially requested on the basis of diagnostic suspicion:

- Genetic tests have to be considered important in atypical cases of difficult diagnosis
- In patients showing autism, intellectual disability, and congenital malformations chromosomal microarray may be useful for a correct diagnosis
- Evaluation by ophthalmologist, cardiologist and clinical geneticist, *FBN1* gene sequencing if Marfan syndrome is suspected
- Karyotype if Klinefelter syndrome or triple X syndrome are probable
- Analysis of *FMR1* CGG repeat number when Fragile X syndrome is considered
- Plasma homocysteine level if there are criteria for homocystinuria
- Bone age, evaluation by clinical geneticist, *NSD1* sequencing when Sotos syndrome is considered.

### Genetics of tall stature

Human growth and adult height are highly heritable (h2 = 0.80–0.90) polygenic traits [28, 29]. Initially, the study of genetics of height mainly focused on short stature, with many possible candidate genes identified. Recently, some studies focused on genetics in extremely tall individuals and found some common genetic variations. Exome sequencing has been demonstrated to be useful for identifying novel genes involved in aetiology of tall stature.

For example, a common polymorphism in the *high-mobility group A2 (HMGA2)* gene was significantly associated with tall stature [30]. Furthermore, overproduction of C-type natriuretic peptide by chromosomal translocation [31, 32] and gain-of-function mutations in *natriuretic peptide receptor-2 (NPR2)* gene have been reported to be responsible for overgrowth syndromes [33, 34] or extremely tall stature without skeletal deformities [35]. Heterozygous los-of-function mutations of *fibroblast growth factor receptor-3 (FGFR3)* gene results in a aphenotype characterized by camptodactyly, tall stature and hearing loss (CATSHL syndrome [36, 37]. Finally, a recent article identified an association of a novel homozygous mutation in *Sec23 homolog A (SEC23A)* gene and a previously reported homozygous mutation in *mannosidase alpha class 1B member 1 (MAN1B1)* gene in two patients, from a consanguineous family of Lebanese origin, presenting somatic overgrowth, macrocephaly, mild dysmorphic features, hypertelorism, maloccluded teeth, intellectual disability and flat feet [38].

In our recent in-press paper, we found significantly higher GH receptor gene expression levels in tall children compared with short and normal statures ones, suggesting that GH sensitivity could be involved in the mechanisms on the basis of tall stature [39].

### Treatment of tall stature

Generally, tall stature is not a pathological condition and does not need treatment.

Children and their parents may seek treatment to reduce growth if they find the predicted adult height unacceptable but treatment is generally only considered for adolescents whose predicted adult height is more than 2.5 SD above the population mean.

Social impact in tall patients may be a cause of anxiety in parents who request treatment to cease growth progression. Specific therapy depends on the prevision of the final height. Various types of treatment, either hormonal or surgical, have been used to reduce growth.

The most accepted and effective treatment for an excessive height prediction is inducing puberty early and leading to a complete fusion of the epiphyses and achievement of final height, using testosterone in males and oestrogens in females.

In males, testosterone enanthate is usually used at the doses of 250–500 mg/ 2 week for 6-9 months. Side effects as aggressive behaviour, painful erections, severe forms of acne and increased risk of developing prostatic cancer may be observed [40].

In females, the most accepted treatment is 17β-estradiol at the dose of 0.2–4 mg/die, adding a progestin (10 mg) for 1 week a month. Treated patients may show weight increase, headache, hypertension, increased risk of thromboembolic events or of developing breast cancer and melanoma [41]. Furthermore, it is important to exclude a mutation of the Villebrand factor gene before start the treatment.

Furthermore, late negative effects on fertility in females have been reported. High dose testosterone in boys is less effective than oestrogen treatment in girls, but the achieved height reduction depends on the bone age at which treatment is started.

It has been reported that a nocturnal infusion of octreotide, a somatostatin analogue (201–995), reduces GH secretion and height prediction in tall children [42, 43]. However, a more recent study, showed that long-term treatment with analogue of somatostatin (201–995) does not reduce final height in a manner sufficient to justify the treatment in tall stature [44].

The most common surgical procedure for reducing growth is bilateral percutaneous epiphysiodesis of the distal femur and proximal tibia and fibula. Epiphysiodesis is a surgical intervention for reducing final height by 5 cm, which is performed at a bone age exceeding 12.5 years in girls with a stature of 170 cm and 14 years in boys with a stature more than 185 cm [2, 45, 46]. The procedure itself is short (about 60–70 min) and patients are allowed to stand on their legs directly afterwards but are advised not to engage in athletic activities for 4 weeks. In experienced hands, few complications are seen.

## Conclusions

The majority of subjects with tall stature show a familial tall stature or a constitutional advanced growth, which is a diagnosis of exclusion. Physicians should always search for a pathological cause of tall stature, although the majority of children are healthy. However, some tall subjects may develop severe complications such as a dissecting aneurysm in early adult life in patients with Marfan syndrome. A careful physical examination and an evaluation of growth pattern are required before starting further investigations.

## Abbreviations

CBS: Cystathionine β-synthase
ERα: Oestrogen receptor α
FSH: Follicle stimulating hormone
GH: Growth hormone
GnRH: Gonadotropin releasing hormone
IGF-I: Insulin-like growth factor-I
LH: Luteinizing hormone
MPH: Midparental height
SD: Standard deviation
SDS: Standard deviation score
TSH: Thyroid stimulating hormone
